# 儿童极重症血小板增多症的临床病因研究

**DOI:** 10.3760/cma.j.issn.0253-2727.2023.04.015

**Published:** 2023-04

**Authors:** 琳 苟, 玥瑶 刘, 超 林, 鸽 张, 举 高, 易萍 朱, 霞 郭, 晓茜 陆, 志贵 马

**Affiliations:** 四川大学华西第二医院儿童血液肿瘤科，出生缺陷与相关妇儿疾病教育部重点实验室，成都 610041 West China Second University Hospital, Key Laboratory of Obstetric & Gynecologic and Pediatric Diseases and Birth Defects of Ministry of Education, Chengdu 610041, China

极重症血小板增多症（extreme thrombocytosis, EXT）指外周血中的血小板计数（PLT）>1 000×10^9^/L[1]–[2]。通常认为绝大多数EXT患者患有克隆性血液系统疾病，因炎症很少对血小板产生如此大的影响，但既往关于EXT的研究表明情况并非如此，尤其对于儿童患者[3]–[5]。本研究探讨儿童EXT的可能病因并通过对血小板极度增高的患儿进行追踪随访和基因筛查，以发现隐匿的肿瘤性疾病的频率。

## 病例与方法

1. 病例：本项回顾性研究纳入2017年1月至2018年12月期间在四川大学华西第二医院就医且符合以下条件的患儿：①至少两次血常规检查PLT>1 000×10^9^/L；②年龄≤16岁；③完成随访。

2. 研究方法：收集、分析所有EXT患儿的病例资料，记录自动血细胞分析仪获得的HGB、WBC、PLT及过去1个月内输血、服用可引起血小板增多药物（糖皮质激素、长春新碱等）、外伤、手术等信息。对纳入的每例患儿进行多病因描述。对于具有多种病因的病例，分析血小板计数的趋势以确定主要病因。

3. 统计学处理：采用SPSS 20.0软件进行数据分析，计量资料以“中位数（范围）”表示，而计数资料则以“频数”和“百分数”表示。采用独立样本Mann-Whitney *U*检验来检验连续数据中性别差异的显著性。以*P*<0.05为差异有统计学意义。

## 结果

1. EXT患儿的基本情况：77例患儿符合纳入标准，男45例，女32例，中位年龄0.95（0.04～15.33）岁，年龄及性别分布见表1。中位PLT为1 073（1 001～2 478）×10^9^/L。患儿诊断EXT时的血常规参数详见表2。所有记录的血液学参数在性别之间差异无统计学意义。

**表1:** 77例极重症血小板增多症患儿的性别及年龄分布［例（％）］

性别	<1岁	1～2岁	2～6岁	6～10岁	10～16岁	合计
男	21（53.8）	9（90.0）	9（52.9）	3（75.0）	3（42.9）	45（58.4）
女	18（46.2）	1（10.0）	8（47.1）	1（25.0）	4（57.1）	32（41.6）

**表2:** 77例极重症血小板增多症患儿血常规参数［*M*（范围）］

组别	例数	PLT（×10^9^/L）	WBC（×10^9^/L）	HGB（g/L）	RBC（×10^12^/L）	PDW（%）	MPV（fl）	P-LCR（%）	PCT（%）	RDW-CV
男	45	1067（1 001~1 541）	11.3（2.7~247.8）	101（71~138）	3.86（2.87~5.59）	9.5（7.2~12.7）	9.1（7.4~10.6）	16.7（6.6~28.9）	0.98（0.83~1.41）	16.2（13.2~37.1）
女	32	1092（1 001~2 478）	12.8（3.8~44.9）	104（75~147）	4.21（2.40~5.59）	9.7（7.6~14.2）	9.1（7.9~10.9）	17.1（10.0~33.4）	0.98（0.82~2.20）	15.5（12.9~24.2）

统计量		−1.091	−1.029	−1.712	−1.861	−0.311	−0.563	−0.453	−0.802	−1.82
*P*值		0.275	0.304	0.087	0.063	0.756	0.574	0.651	0.422	0.069

**注** PDW：血小板体积分布宽度；MPV：血小板平均体积；P-LCR：大血小板比率；PCT：血小板比积；RDW-CV：红细胞体积分布宽度变异系数

2. 病因：71例（92.2％）患儿为反应性血小板增多症，3例（3.9％）为克隆性血小板增多症，3例（3.9％）两种病因并存。其中，49例（68.8％）EXT患儿为多种病因，其余24例（31.2％）患儿仅确定了1个病因。对于多因素导致的EXT病例，我们根据血小板计数的变化趋势以及当时的临床事件确定了每个病例的主要病因，感染（50.6％）是主要原因，其次是药物（15.6％）和贫血（11.7％），详见表3。

**表3:** 77例极重症血小板增多症的主要病因

病因	例数（%）
克隆性血小板增多症	
原发性血小板增多症	4（5.2）
慢性髓性白血病	2（2.6）
继发性血小板增多症	
感染	39（50.6）
药物	12（15.6）
贫血	9（11.7）
川崎病	6（7.8）
无脾	2（2.6）
非血液系统恶性疾病	2（2.6）
炎症性疾病	1（1.3）

3. 病因组合：77例EXT患儿的病因组合根据血小板计数趋势以及当时的临床事件确定并分类。感染（50.6％）是EXT患儿的最常见的主要病因，其中最多的是感染并发贫血（14.3％），其次为仅感染（11.7％）和感染合并药物（11.7％）。药物（15.6％）是12例EXT患儿的主要病因，均是肿瘤化疗药物导致的骨髓抑制后血小板反跳性增高，其中以急性淋巴细胞白血病居多，均伴随感染和（或）贫血。9例EXT患儿的主要病因是贫血（11.7％），其中8例患儿仅贫血，1例贫血伴感染及药物。川崎病（7.8％）和血液系统肿瘤（7.8％）也是EXT患儿的常见主要病因，而无脾（2.6％）、非血液系统恶性疾病（2.6％）和炎症性疾病（1.3％）很少为EXT患儿的主要病因。

4．新诊断的肿瘤性疾患：8例EXT患儿（10.4％）被诊断为新发肿瘤，包括6例（7.8％）血液系统肿瘤和2例（2.6％）非血液系统肿瘤。血液系统肿瘤中最常见的是原发性血小板增多症（ET）（66.7％），其次是慢性髓性白血病（CML）（33.3％）。2例新诊断非血液系统恶性肿瘤均为肝母细胞瘤。

## 讨论

根据病因，血小板增多症可分为克隆性（原发性）和继发性（反应性）两类。原发性血小板增多症是一种骨髓增殖性疾病，由造血细胞的单克隆或多克隆异常或促血小板生成素（TPO）生物学异常引起[1],[6]。继发性血小板增多症是在各种血液学或非血液学病因，如特发性关节炎等炎症性疾病、感染或肿瘤性疾病等情况下，血小板生成素、白细胞介素-6、以及其他细胞因子或儿茶酚胺内源性水平升高造成的刺激性巨核细胞生成而引起的[7]。血小板计数升高的程度不能明确区分克隆性和反应性血小板增多症。

本研究是目前规模最大的关于儿童EXT的病因研究。与之前EXT患者单一病因研究不同，且既往研究对象绝大多数为成年患者，我们调查了儿童EXT发作期间存在的几乎所有潜在致病因素。在大多数情况下（68.8％），EXT可以被认为是多病因的,血小板的极度升高可能是来自各种已知的病因累积“命中”的结果。

在本研究中，77例EXT患儿大多数为<1岁年龄组（50.6％），这与之前针对血小板增多症患儿的研究一致。在以往研究中，血小板增多症在2岁以下的婴儿和儿童中更常见[8]–[9]。新生儿期对血小板增多症的易感性较高可能源于骨髓中TPO基因表达高，循环TPO浓度高于儿童和成人，巨核细胞祖细胞对TPO的敏感性增加等原因[10]。在所有年龄组中，观察到EXT在男童（58.4％）中比在女童（41.6％）中更常见，另外几项研究也观察到类似的结果[8]。临床和分子证据表明，性激素（尤其是雄激素）可介导血小板数量和功能[11]。在体外，雄激素可增强花生四烯酸诱导的人血小板聚集，雄激素治疗可提升骨髓增生异常和血小板减少症患者的血小板数量；在小鼠中，去势可减少血小板生成，而睾酮可恢复血小板生成[12]–[13]。但我们并没有观察到EXT患儿的血液学参数的性别之间的差异无统计学意义。

我们总结了每例EXT患儿的主要病因，并根据主要病因对患者进行了分组。感染和药物是儿童EXT的2个最主要的原因。这和既往关于成人EXT患者研究报告的手术并发症和血液系统肿瘤是EXT的2个最常见原因不一致[14]。与之前报道的EXT与危重疾病有关的研究相似[15]，我们发现在以感染为主病因的EXT患儿中，重症肺炎和脓毒症的发病率较高，分别为35.9％和41％，这提示医生在诊治提示有感染病因的EXT患儿时需警惕重症感染和脓毒症的发生并及时干预。化疗药物、以及类固醇等药物能引起血小板升高。其中，化疗药物如抗有丝分裂药（阿霉素/多柔比星、柔红霉素、博来霉素）、抗代谢药（阿糖胞苷）和烷化剂（顺铂、卡铂和异环磷酰胺）通过抑制骨髓使血小板反跳性增高；而糖皮质激素通过将储存的血小板从脾脏释放到血液循环中，可导致短暂性血小板增多[16]。12例以药物为主要病因的EXT患儿均是患肿瘤后接受了化疗药物，导致骨髓抑制后血小板反跳性增高，其中以急性淋巴细胞白血病最多。而糖皮质激素常作为伴随因素而非主要病因，在以感染为主病因的患儿中同时出现。

本研究在既往文献对EXT成人患者病因分类的基础上[14]，增加了川崎病这一儿童特有病因。川崎病是一种中血管血管炎，易引起冠状动脉异常[17]。川崎病患者中一直有血小板增多症的报道，在疾病的第2、3周经常见到[17]–[18]，最初被认为是继发于潜在炎症的良性反应性现象，但近年来有研究表明血小板数目以及血小板活化可能是与川崎病相关的各种并发症的主要决定因素[18]。在6例以川崎病为主要病因的EXT患儿中，只有1例患儿在住院期间发现冠状动脉扩张，是否与血小板的极度升高有关仍需要进一步研究。

在大多数血小板增多症患儿中，无论有活动性病变，还是有潜在性疾病，他们常有明显的临床症状，易于诊断。然而，在其他情况下，亚临床疾病（例如隐匿性癌症）可能是血小板增多症的原因，容易漏诊，这对临床医生提出了更棘手的诊断挑战。在我们的研究中，新发肿瘤性疾病8例，其中6例为骨髓增殖性肿瘤（MPN），2例患儿为非血液系统恶性肿瘤，且都为肝母细胞瘤。在6例MPN患儿中，4例为ET，其余2例为CML。ET患儿通常以长时间的血小板居高不降为主要特征，无其他主诉，这提示在无法找到明确病因的持续性的血小板极度增高时高度提示ET。2例肝母细胞瘤患儿都有腹部包块体征，这提示医生在门诊为EXT患儿查体时应注意腹部体征。在我们的随访中，1例肝母细胞瘤患儿由于肿瘤病情死亡，不被视为EXT的直接结果。

本研究有如下局限性：①本研究是在三级甲等儿童专科医院（无小儿外科）进行的，转诊偏倚是无法避免的。在本研究中，重症感染、血液系统恶性肿瘤患儿的比例可能高于社区环境的预期，手术患儿的比例较低。②纳入标准要求病例至少有2次血液常规检查以尽量减少实验室误差并显示持久性，排除了只有1次血常规检查提示EXT的患儿，具有短暂性EXT或失去随访的患者未被纳入研究。

综上所述，儿童EXT是一种多病因疾病，感染是最常见的主要病因。当没有其他病因可寻时，隐匿的恶性疾病是需要临床医生纳入考虑的，应该定期随访，必要时做基因筛查。
